# IDeS + TRIZ: Sustainability Applied to DfAM for Polymer-Based Automotive Components

**DOI:** 10.3390/polym18020239

**Published:** 2026-01-16

**Authors:** Christian Leon-Cardenas, Giampiero Donnici, Alfredo Liverani, Leonardo Frizziero

**Affiliations:** Industrial Engineering Department, AlmaMater Studiorum Università di Bologna, 40126 Bologna, Italy; giampiero.donnici@unibo.it (G.D.); alfredo.liverani@unibo.it (A.L.); leonardo.frizziero@unibo.it (L.F.)

**Keywords:** additive manufacturing, product reverse engineering, IDeS, TRIZ, product design

## Abstract

This study aims to gather a sustainable understanding of additive manufacturing and other Manufacturing 4.0 approaches like horizontal and vertical integration and cloud computing techniques with a focus on industrial applications. The DfAM will apply 4.0 tools to gather product feasibility and execution, with CAE—FEM analysis and CAM. This publication focuses on the redesign of a vehicle suspension arm. The main objective is to apply innovative design techniques that optimize component performance while minimizing cost and time. The IDeS method and TRIZ methodology were used, resulting in a composite element, aiming to make the FDM-sourced process a viable option, with a weight reduction of more than 80%, with less material consumption and, hence, less vehicle energy consumption. The part obtained is holistically sustainable as it was obtained by reducing the overall labor used and material/scrap generated, and the IDES data sharing minimized rework and optimized the overall production time.

## 1. Introduction

Product development in the modern era has led to an increase in technological complexity, along with the opportunity to leverage it with connectivity and centralized data sharing, helping to lower the overall product environmental footprint. This data commonality allows us to create a sustainable and user-centric design. Here are some key trends and features that have shaped product development in this era, starting from Rapid Technological Advancements with an exponential growth in technology functionality and dependability, particularly in the fields of computing, artificial intelligence (AI), Internet of Things (IoT), and data analytics. These technological advances have enabled the creation of smarter, more interconnected products that can collect and analyze data to offer enhanced user experiences and improved functionality. Afterwards, in terms of the internet and connectivity, the widespread availability of the internet and the proliferation of connected devices gave rise to the concept of the Internet of Things (IoT) [1]. Products are now designed to be interconnected, allowing users to control and monitor them remotely through smartphones and other devices. This has opened up new opportunities for creating products and services with seamless integration and real-time communication [2].

Likewise, companies have evolved their practices towards the Industry 4.0 era by adopting Agile Development procedures, in contrast to traditional waterfall product development. Agile Development allows for incremental and flexible product iterations, enabling faster responses to changing market demands and reducing time to market [3]. This approach fosters collaboration among cross-functional teams and prioritizes frequent customer feedback. In addition to data-driven decision making, with the abundance of data available, product development teams rely on data-driven decision making. By analyzing user data and product usage patterns, companies gain insights into user behavior, preferences, and pain points, allowing for more informed and effective product improvements.

In addition to this, industries can exploit innovations driven by technology for the benefit of more stakeholders, with companies from different sectors collaborating and integrating technologies to develop innovative products. For example, the convergence of healthcare and technology [4] has led to the rise in wearable health devices, telemedicine solutions, as well as important improvements in orthopedics [5,6,7] and transplants, by using technology inspired by the aerospace industry. This unified platform and ecosystem product approach will help companies to create products designed to integrate seamlessly with other complementary products and services. This approach creates more value for users, enhances customer loyalty, and opens up new revenue streams.

A number of product development tools have been released, offering ways to mitigate these risks, and many of them fall under Lean Product Development (LPD). Conversely, some of these tools are taken by mainstream product development, whereas others have not been fully implemented or tried at the industry level. The premise of a product development tool and method that improves the efficacy of the development process has long been promoted. However, many of these tools have failed to be applied at the company level [8]. This involves the need to enable each area of the organization to accurately understand customer-related data. Consequently, an organization could attract and retain the most valuable portion of the market by creating value for the customer; this would directly impact the entire chain, generating value that can later be related to approaches on social responsibility [9]. This statement was also confirmed by [10,11,12] who also underline the importance of taking into account each stakeholder across the entire value chain creation for better results in a cross-functional organization.

### 1.1. IDeS Method Towards Achieving Smart and Sustainable Product Engineering

**Figure 1:**
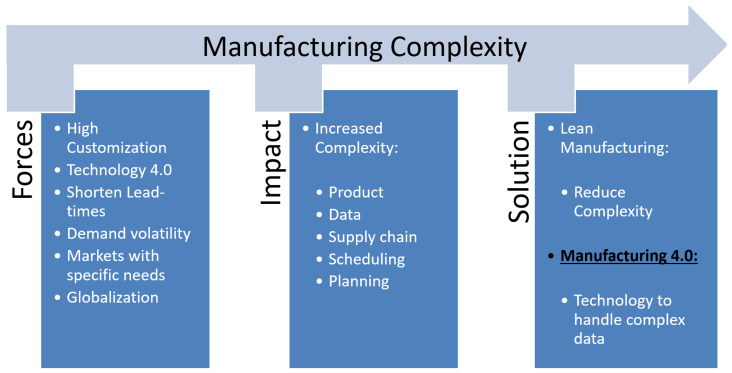
Manufacturing Complexity Assessment and 4.0 tools approach.

Moreover, smart-driven solutions have revolutionized the way in which a product is conceived altogether, first to understand new market behavior and heading towards individualized products, using data analysis flow across the entire production chain to portray data-inspired solutions that help organizations to suit their infrastructure in a flexible, more efficient manner. Additionally, companies are moving to adopt customer-driven processes by creating new procedures that adopt the will of every customer [13,14]. Other analyses by [15,16,17] established that nowadays the volatility of the customer behavior has turned the individual customer orientation concept into a more objective way for market analysis, and differentiating or customizing a product or service is useful to even obtain extra information about the customer’s maximum necessities. Therefore, increased data analysis capacity in tools from Industry 4.0 has enabled companies to develop complex evaluations to gather as much detailed information [18] that can provide optimal solutions to challenges with the integration of tools for Manufacturing 4.0 (Figure 1).

Nowadays, accurate information management across the automotive industry is one of the key issues in the industry towards sustainability, as it is suffering from the most basic issues expected from an area that employs people and whose industry is valued at USD 2.6 tr in 2023 [19], which is an interesting 10 percent over-year decrease from 2.86 trillion U.S. dollars, as of 2022 [20]. An accelerating industry-changing scenario leaves companies without many resources to modernize internal data management platforms [21]. This old-fashioned data interchange and integral project auditing is starting to deliver its first reactions [22]. Longer times of product development and delivery at a premium price are because of highly structured system complexities of their own. These technical and management problems have then been translated into longer deliverable processing times, therefore appearing to cause many management issues that could be decreased with the adoption of a unified platform. Platform integration across global companies has been demonstrating an undoubted need to adopt a unified central data interchange policy, which ought to obtain a leaner, more efficient use of resources [23]. Companies nowadays exploit the infinite amount of data that will make better use of resources, driving sustainability and flexibility in the industry.

Moreover, the Industry Design Structure Method (IDeS) establishes the creation of continuous feedback links between the design structure with the other departments related to product development across the organization. Likewise, the main three macro-phases of this concept summarize the entire industrial product design process (Set up, Development, Production). Consequently, the main IDeS output is a customer-centered, technically and demand-wise secured product design company organization. This is obtained by structuring the organization transversally with quick adaptation to today’s industrial challenges. Being able to train new professionals in the skills required for obtaining good results with Manufacturing 4.0 in the company was achieved by joining all organizational departments from product developers to manufacturing and quality control areas. In the end, this information would improve an organization’s effectiveness in maximizing consumer profitability [24] from the traditional, known methodologies centered on product effectiveness, as previously introduced by [25]; this evolution has to be carried over the entire value chain [26] until redefining the sales structure [27]. Therefore, a modern organization structure is established that would interactively update the knowledge and analyze it to forecast the specific requirements that would deliver exclusive value to its users.

Furthermore, some applications of the IDeS method towards product differentiation with tools like Stylistic Design Engineering (SDE) [28,29] and TRIZ aid in choosing the best product packaging combination, in line with the design specifications (volumes, geometries, maximum allowed overall dimensions, and so on).

With this, the product development macro-phase would include the final CAD design of the product, good to be verified by means of virtual finite element simulation tests, and virtual and initial physical prototyping of the product or some of its parts takes place. This is followed by rapid prototyping and experimental testing phases for structural validation of each component. Industry 4.0 will use this data, together with the production line information, to design a manufacturing setup that best meets the production targets [30]. After that, the product BOM will be fully defined, and the organization of the manufacturing process and the first series prototypes of the product can proceed.

**Figure 2:**
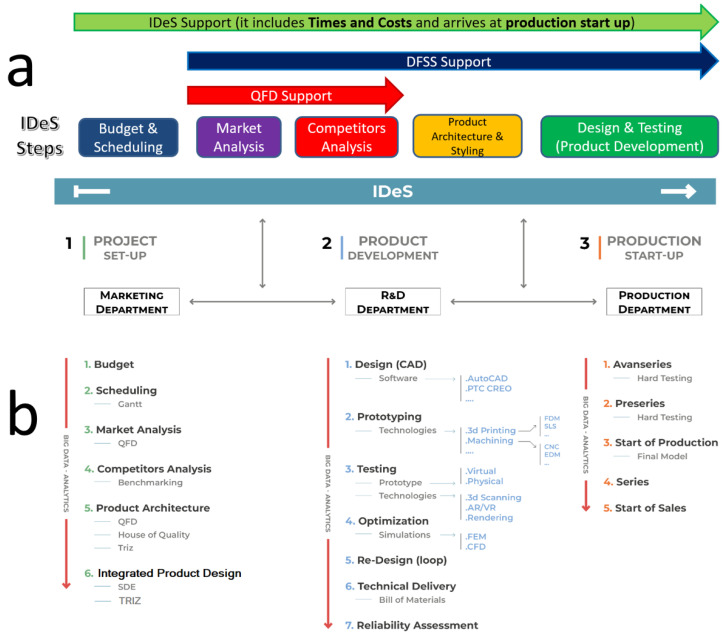
(**a**). IDeS method integrated from QFD and DFSS; (**b**). IDES method deployment: tool overview.

Consequently, the IDeS method has proven to be able to track down the entire development stages of an industrial project, starting with the birth of the idea, from a “white sheet” project definition, then adding up Budget and Scheduling targets, towards a market definition and positioning, continuing with product and process engineering until the Start of Production (SOP) milestone [31]. In this way, IDeS has demonstrated the ability to embrace wide organizational management and traceability, integrating product design quality-driven tools like QFD and Design for Six Sigma (DFSS) [32] as seen in Figure 2a.

However, overall product lightweighting is a top priority in the automotive industry, to guarantee a lower vehicle environmental footprint [33,34,35]. The industry has proposed the use of 4.0 technologies to address this challenge with methods to properly apply composite materials and create structurally efficient products. This Design for AM has demanded to exploit the most well-known methods for virtual and physical product validation [36,37] when using composite materials and complex part structures [38,39]. The latter will add up to big data analytics that use digital part data for quality and flexible manufacturing parameters [30,40].

Moreover, optimizing a vehicle’s suspension arms means improving its performance and weight. Suspension arms are mechanical elements that connect the wheels to the vehicle chassis and influence their stability, handling, and comfort. The aim of suspension arm optimization is to find the most suitable shape and material to maximize the strength, stiffness and lightness of the arms while reducing costs and environmental impact.

### 1.2. Case Study

**Figure 3:**
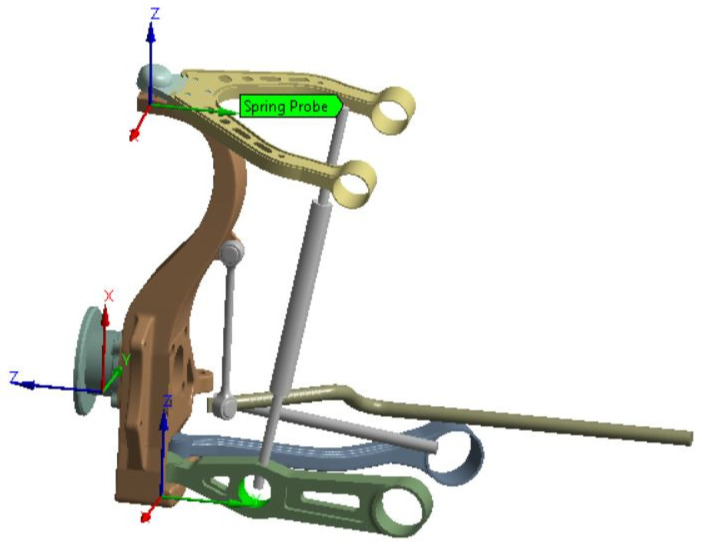
CAD model of the left front suspension, used for dynamic analysis on Ansys.

The case study concerns the re-engineering setup of the front suspension arms of the Tesla Model S (Austin, TX, USA), a high-performance electric car. The objective is to improve the dynamic and structural characteristics of the arms by reducing weight and defining a geometry that can exploit the advantages of AM. The suspension under investigation is of the ‘double wishbone’ type [Figure 3].

This type of suspension keeps the effects of the left and right wheels on the same axle separate. In this way, the study can focus on only one half of the suspension, simplifying the treatment and the related calculations.

The subsequent considerations, calculations and models are based on the CAD model in Figure 3, which has been simplified by separating it from the entire assembly. This simplification concerns the connections between the joints without the modeling of the bearings, which does not consider friction. A further simplification concerns the structure of the steering element, which is modeled with a simpler geometry.

Finally, the research challenges of this study would use a systematic methodology that integrates customer needs (QFD), inventive problem solving (TRIZ), and a full product development pipeline (IDeS) to re-engineer a load-bearing automotive component and reach the improvement targets. Product engineering uses the I4.0 methodologies to reengineer and optimize the part to achieve the best performance, with a focus on manufacturing process (AM) and quality control using 3D scanning. When reengineering a product, design methodologies (such as Design for AM) including internal macrostructure design and novel material mixes should be considered. Additionally, high-performance components with intricate geometries that are unattainable with traditional technologies, as well as customized and structurally optimized parts with minimal material use, can be produced with additive manufacturing (AM).

## 2. Materials and Methods

The IDES approach with technical focus will exemplify the use of the same tools and techniques given in early released studies of this methodology in order to assess the feasibility of conveying a technical solution, given a set of constraints and targets to achieve.

The three main targets to achieve are as follows:

- Less energy consumption of the vehicle.
- Contribute to reducing polluting CO_2_ emissions target for Europe by 2035.
- Exploits manufacturing technologies given by Industry 4.0.

The first part of this study will examine the layout of the front suspension of a current-production vehicle with the objective of evolving the current-production parts with reengineered ones that allow the overall system to save weight, hence contributing to vehicle emissions reduction, and overall energy consumption of the vehicle.

The second part of this study portrays the complete methodology to seamlessly produce industrially acceptable elements sourced with composites and AM, whilst these elements were originally engineered to be built with traditional materials and methods for processing them. The following steps were outlined in order to pursue the main objective.

1. Starting up input source element data: geometry, materials, and mechanical requirements.
2. Definition of supply materials, processes for best efficiency.

(a) Optimize the part geometry for best results with new technologies to implement (AM, carbon fiber).
(b) Proper Materials Specification definition > with technical data sheets and performance tests.
(c) Printing equipment details for best quality.

3. Optimization of the operating parameters for all processes of step 1, simulations of the optimized part to freeze the model.
4. Development of the physical part.
5. Part quality control assessment.

**Figure 4:**
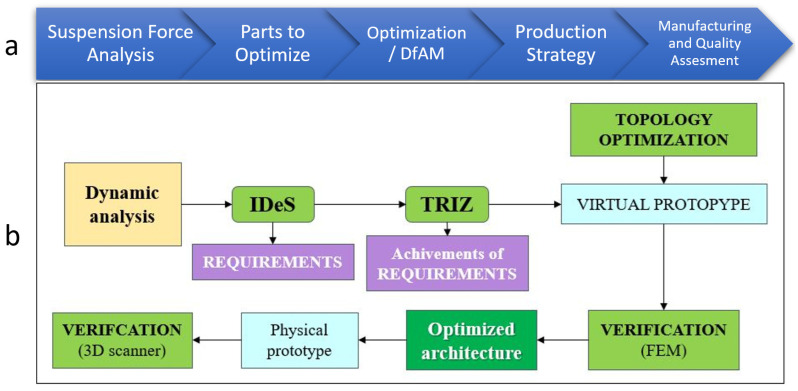
(**a**). IDES product design method; (**b**). method applied to DfAM.

The overall IDES approach to the product design process can be seen in Figure 4a, whilst the study workflow can be summarized with a block diagram [Figure 4b] in which each block represents a step performed.

The application of the methodology was given by the re-engineering of an automotive part in order to assess its viability to be produced with composite materials by means of additive manufacturing and carbon fiber lamination, as well as using state-of-the-art quality control processes. The aim is to use an existing automotive component already in production with traditional materials, in order to redesign, test, manufacture and assess the viability of using new manufacturing technologies for manufacturing such parts.

**Figure 5:**
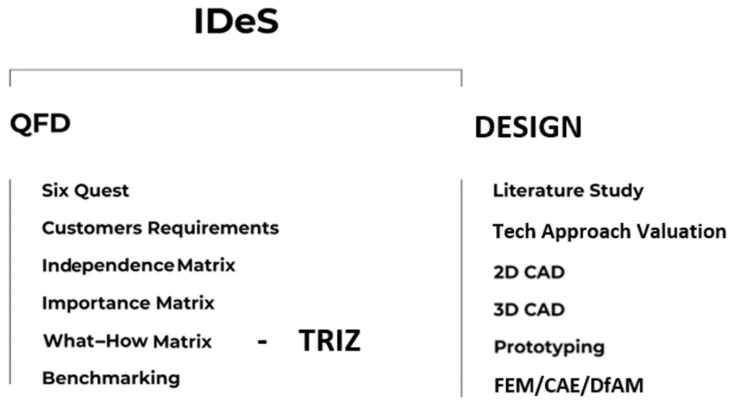
IDeS tools chosen for this study.

For the design of the suspension arms, we started by following proper tools chosen from the methodology IDeS (Integrated Design System) approach [Figure 5]. In particular, the focus was on the QFD (Quality Function Deployment) analysis, from which the characteristics that the optimized suspension arms should have been highlighted. The first focused on the analysis of the six questions (Who, What, When, Where, Why, How) to define who the stakeholders are, what they want from the product, when they use it, where they use it, why they use it and how they use it. From this analysis, characteristics emerged that the suspension arms should have, such as the following:

- Lightness.
- Speed of realization.
- Ease of realization.
- Innovation.
- Mechanical resistance.
- Fatigue life.
- Aesthetics.
- Simplicity of design.
- Component versatility.
- Customization.

From these requirements, we constructed an independence matrix, which shows the degree of dependence among the criteria, and an importance matrix, which expresses the relative weight of each criterion. Finally, we used a selection algorithm that compares alternatives based on their performance on the criteria and their independence. The result is a set of requirements that maximizes stakeholder satisfaction and minimizes conflicts between criteria:

- Lightness.
- Ease, Precision of fabrication.
- Mechanical strength.
- Fatigue life.
- Simplicity of design.
- Productivity.

Therefore, product designers took these main concepts and developed them into feasible engineering targets to achieve the objectives outlined. Lattice Structures were chosen as a method to achieve lightness, mechanical strength and fatigue life. Then the proper choice of AM method would rule out towards manufacturability precision, productivity, aesthetics and design complexity.

TRIZ

The TRIZ method of identifying and resolving conflicts between technical requirements and available resources was followed to find practical solutions that indicate how to achieve these requirements in the components to be made. The requirements considered are those found from the QFD analysis. These were interpreted using the parameters of TRIZ, and through the Matrix of Contradictions possible solutions of a general nature were found, identified among the 40 inventive principles of TRIZ. The solutions found are the following:

- Composite materials.
- Porous materials.
- Economic objects.
- Replacement of mechanisms.

These solutions were interpreted to generate several possible solutions and evaluated according to the selection criteria. The solution adopted took all four proposed solutions to make the skeleton of the component from carbon fiber polymer material by FDM printing on which a layer of composite material (carbon fiber) was applied, with the purpose of reinforcing the overall structure. With this, an economic replacement of mechanisms was performed to achieve a porous structure (lattice) created with composites.

**(A) QFD—To find out technical characteristics.**

To evaluate the best characteristics of an innovative product, it is necessary to study dependency relationships existing between the customer requirements. The QFD method was used to achieve this goal. This method makes use of matrices of interrelation, which provide the tools to create a unique ranking between the requirements. The first step is the individuation of these requirements using six basic questions: who, what, where, when, why, and how. Having obtained all the requirements, it is then possible to move on to the interrelation matrices.

The analyses were carried out using the QFD method. Moreover, the methods of QFD analysis used while carrying out this project were as follows: the six questions, the relative importance matrix, the independence matrix, and the what–how matrix, in addition to the benchmark and the top-flop analysis. They were analyzed individually.

**(A1) Six Questions and Overall Requirements**

The six questions of the QFD were applied to the case study in the first design phase of the innovative product. The answers, which allow the requirements from analyses with the interrelation matrix to be found, are crucial in the next analysis. From the preceding analysis and the research carried out, keywords were extrapolated for the product to be designed, which serve as a guideline in the subsequent matrix analysis and design phases.

**Figure 6:**
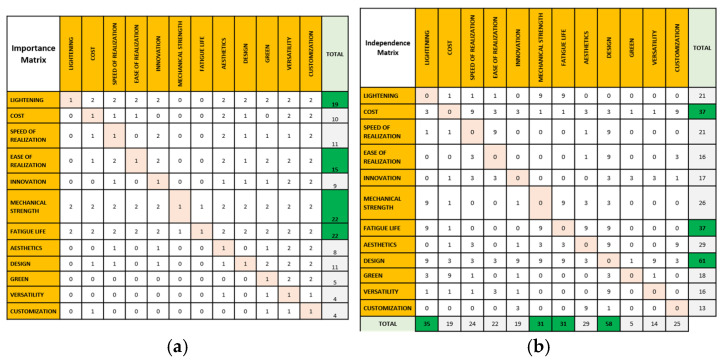

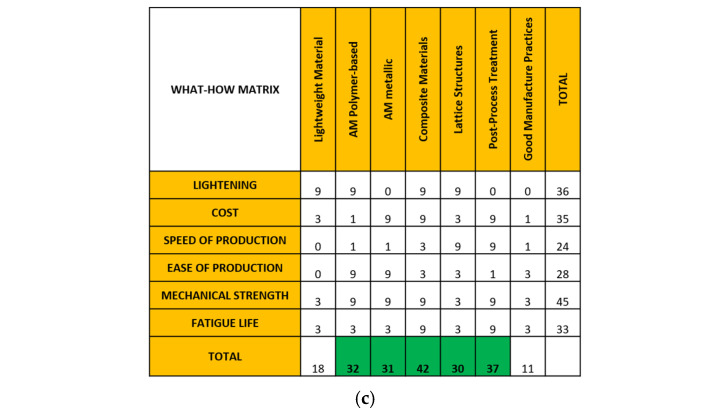
(**a**) Indipendence Matrix; (**b**) Importance Matrix; (**c**) What–How Matrix.

Answers to the six-question analysis made it possible to find some keywords that were used to narrow the field in the next analysis in Table 1:

**(A2) Independence Matrix (Figure 6a)**

The independence matrix was built by placing all the Overall Requirements items, obtained from the six questions, both in columns and in rows. The goal is to determine the cause-and-effect relationships for each parameter by asking the question: how does the element in the row depend on the elements in the columns?

Numeric values, distinguishing between null link (empty box), weak (1), medium (3), or strong (9), were entered in the matrix to quantify the dependence relationship between each column and row element. The independence matrix (Figure 6a) puts requirements found in the previous analysis on a scale of importance. The most important elements for the designer in the preliminary stages of design are summarized in the characteristics stated in the rows and columns of the chart. Then an interpolation of these features was performed based on the factual needs regarding the internal system and the final user; the values in the chart were identified through a scoring system agreed upon upstream of the analysis.

Therefore, the proper characteristics of rows and columns were identified after answering the six questions, which identified the most important characteristics to be used for this project.

**(A3) Importance Matrix (Figure 6b)**

While the construction is similar to the previous matrix, a different question was analyzed: is the element in the row more important than the element in the column?

As in the matrix described above, the numerical values are distinguished in

1: The row element and the column element have the same importance;

0: The row element is less important than the column element;

2: The row element is more important than the column element.

The importance matrix (Figure 6b) narrows the field even more to reach more precise innovative characteristics.

**(A4) What–How Matrix (Figure 6c)**

Through what/how matrix, the customer’s requirements were compared with the technical requirements necessary for the realization of the product.

In this case numerical evaluations were used to identify the type of relationship:

Nothing (empty box, equivalent to 0).

Weak (1).

Medium (3).

Strong (9).

The what–how matrix (Figure 6c) is a very useful tool to understand the most influential spec that meets the requirements.

Finally, the functions obtained from the QFD analysis:

*Lightweight Material* *AM Polymer-based* *AM Metallic Composite-Materials*

*Lattice Structures*  *Post-Process Treatment*  *Good Manufacturing Practices*

**Figure 7:**
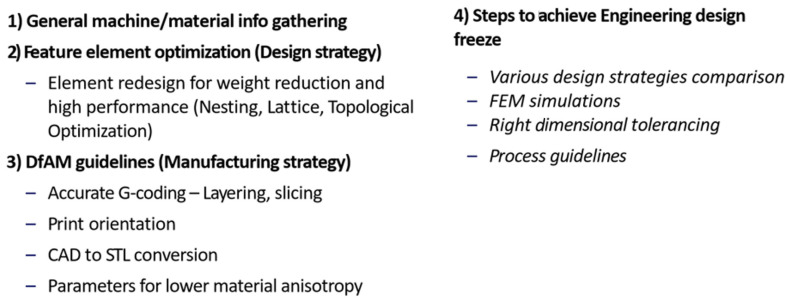
Design for additive manufacturing guidelines.

For ease of the application and analysis of composite materials, as well as arriving at preventive actions, preliminary assessment and achieving a first-hand design feasibility, which allow us to optimize the design straightforwardly, the use of state-of-the-art CAD, CAE, and FEM applications is mandatory. The achievement of the proper internal structure was also achieved by arriving at a proper design for additive manufacturing (DfAM). Guidelines for the application of DfAM are in Figure 7.

Moreover, IDeS method enables organizations to practically implement proper Industry 4.0 technological tooling systematically across all product development areas. This process allowed to take a lower time and resources to perform changes. This updated approach based on the systematic use of technology for product development management enables organizations to widen their product efficiency and minimize the risks.

### 2.1. Method Layout

#### 2.1.1. CAD Model

**Figure 8:**
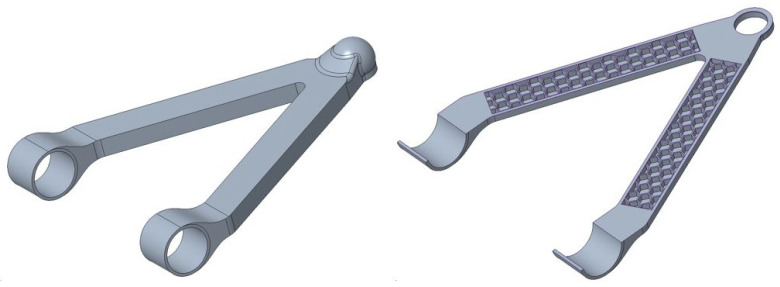
Upper arm optimized version.

Following the indications obtained from topological optimization and taking up the results obtained from the TRIZ methodology, it was possible to construct an initial virtual prototype of the arm. Specifically, for the upper arm, a component with more rectilinear shapes was developed, having a larger resistant section near the joints and a hollow section filled with cellular structures in the less stressed areas, all while maintaining symmetry between the left and right parts, and, similarly, the areas near the joints will be greater than those farther away as they are less stressed [Figure 8].

#### 2.1.2. Materials and Technologies

The next step after making the CAD models of the components [Figure 8] is to decide for each of the arms the material with which the physical prototype will be made. The material must be suitable for fabrication by additive manufacturing but at the same time be strong enough for the stresses acting on the component.

For the upper arm, loaded with moderate forces, a polymer material was chosen: a PETG filament reinforced with 20 percent carbon fiber. Fused Deposition Modeling (FDM) printing technology was chosen to simplify fabrication: this technology offers significant efficiencies over competing technologies, allowing for much lower production costs. It also features good accuracy and high reproducibility.

Next, to verify the components, it is necessary to analyze the stresses to which the components are subjected by finite element analysis. To set up the simulations, it is necessary to know the behavior of the material; although components made by additive manufacturing exhibit anisotropies, it was chosen to model the material as isotropic and to increase the loads according to an appropriate safety coefficient that takes this approximation into account.

This choice is motivated by the fact that modeling a material with the correct anisotropies would require several mechanical tests to characterize the engineering constants, which would be time-consuming and expensive. With the use of a safety coefficient, it was possible to consider the material isotropic, drastically reducing the complexity of modeling.

#### 2.1.3. Dynamic Analysis

The first step is to find the forces acting on the arms by setting up a dynamic analysis of the suspension. The dynamic analysis was carried out using the Ansys Workbench software 2020 R1 on the CAD model shown in Figure 8.

The load was applied on the wheel hub, and, with the appropriate modeling of the joints between the suspension and the chassis, the binding reactions between the arms and the spindle were calculated.

The loads applied on the hub were derived from an analytical calculation of vehicle dynamics considering the vehicle as a rigid body, in which the constrained reactions between the tire and the ground are calculated under different conditions: stationary vehicle, vehicle under braking, vehicle cornering and vehicle under acceleration.

**Figure 9:**
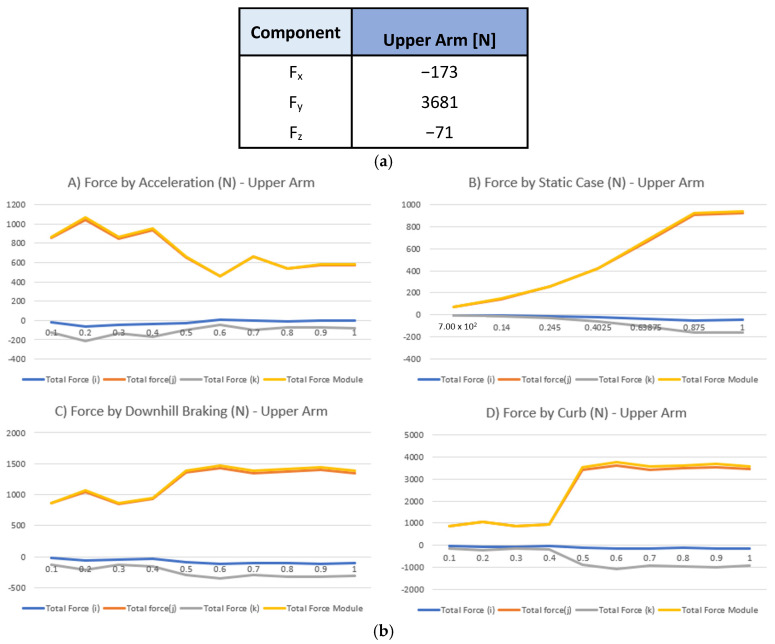
(**a**) Component forces for load case analysis. (**b**) Results on dynamic 4-case analysis on upper arm and stub axle.

From the dynamic analysis [Figure 9b], only the largest values of the components were considered, thus representing all analyzed cases with one model [Figure 9a].

#### 2.1.4. Finite Element Simulation

The advantage of using finite element simulation is that the virtual prototype (CAD model) can be used and can be iteratively modified downstream of the simulation as needed, resulting in significant time savings in design. A disadvantage, on the other hand, is the impossibility of having a reproduction that is identical to reality; the reproduction will be as close as the modeling of the system has been accurate (definition of the material, of ways of applying loads and constraints). Any inaccuracies will significantly affect the results.

The safety coefficient adopted for the simulations is different between the two components because the materials and fabrication technologies are also different.

For the upper arm, a coefficient value of 2 was adopted: this is to take into account the non-negligible anisotropy of the material made for FDM printing as well as the inherent criticality of polymeric materials compared to metallic ones.

**Figure 10:**
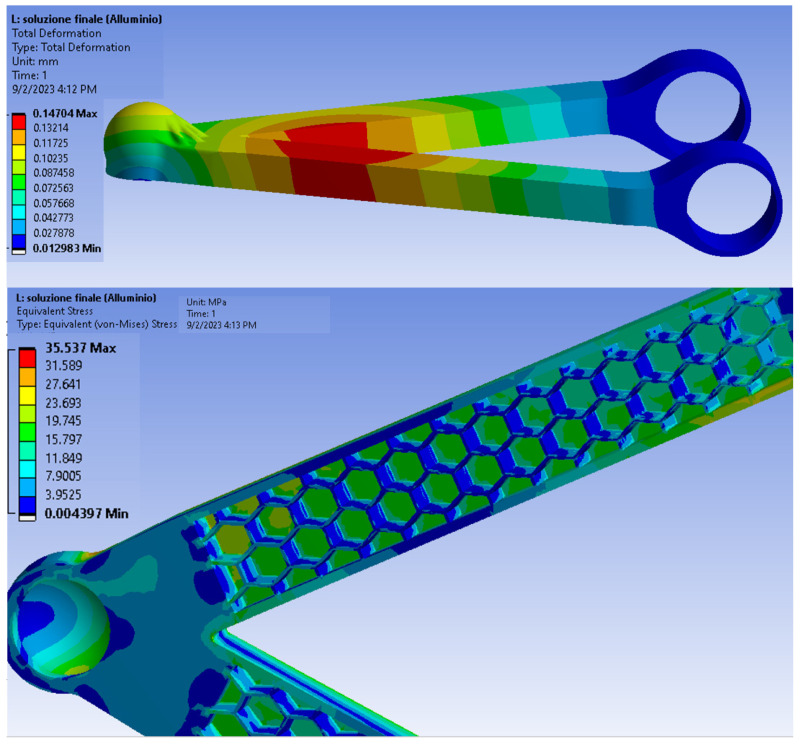
Final iteration with lighter structure and Honeycomb TO and modified Ball Joint geometry; Max Stress = 35.537 MPa; Def Max = 0.15 mm; calculated Life of 1 × 10^8^ cycles min, and a minimum Safety factor of 3.65.

The safety coefficient for finite element simulations is a magnification factor of the loads acting on the components. The results of the finite element analyses performed with Ansys are shown in Figure 10.

## 3. Results

**Figure 11:**
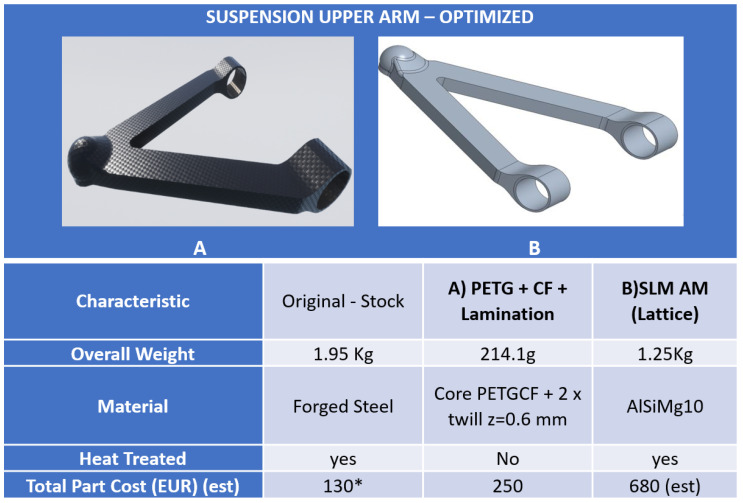
Lightened part in AM versions, carbon fiber PETG (**A**) and SLM (**B**) and key technical achievements. * Production tooling cost not accounted.

As can be seen in both cases, the stresses are acceptable because they are well below the yield stress. In addition, Ansys allows the evaluation of the fatigue life of the component by providing information on the alternating load modes (maximum and minimum stress). For the upper arm the fatigue limit is 100.000 cycles. The reduced value of the fatigue limit for the upper chord is due to the use of a polymer material that is inherently less resistant than the metal material despite the reduced stresses. For the prototyping of the upper arm, the solution made of polymeric material (PETG reinforced with carbon fiber) is considered and compared with the previously calculated SLM AM [Figure 11]. The proposed solution achieved an 85% weight reduction. The significantly higher cost of the SLM process is driven by the high initial investment cost for the tooling and equipment, in addition to operating and post-processing expenses linked to that method.

Furthermore, the prototype was created using the equipment and materials available in the market, and with the aim of saving processing (and post) times for better efficiency, so the part was produced in the same work cell with a single machine, using the PETG-CF filament from Formfutura^®^ (Amsterdam, The Netherlands). The FDM equipment used was the Artillery Sidewinder X1^®^ (Hong Kong, China). The production of the 1:2 scaled part took 12.2 h, for which a 1:1 scaled part with similar build parameters will take about 36 h. The higher build time came after the optimization process, as a correct compromise must be given between part build time and overall material build quality, and the optimized Printing parameters were applied. This prototype did not include the metallic inserts on the bushing’s connectors, but it is recommended to include to portray production intent and prototype testing.

The obtained component geometries were found to be suitable according to finite element simulations. The first step is the definition of the printing plane. This step is critical because it defines the printing direction as well as the direction in which the component will have lower resistance (process anisotropy). Since the loads acting on the arms are planar, the printing plane chosen is parallel to the plane on which the loads are acting.

**Figure 12:**
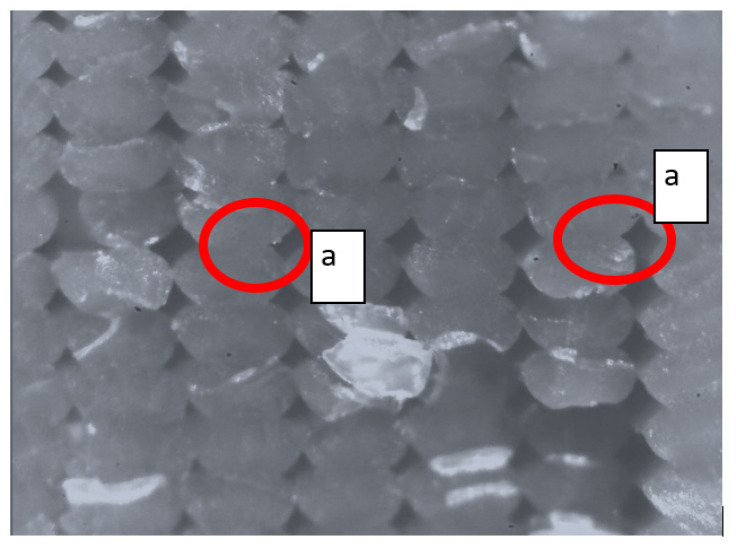
Defect appearance on a PLA specimen: (a) material voids between adjacent lines (red circles).

**Figure 13:**
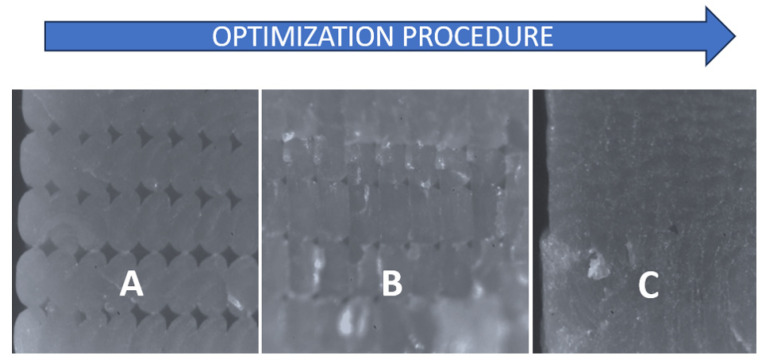
Printing quality microstructures: (**A**) not optimized; (**B**) increasing performance and (**C**) fully optimized.

A proper printing strategy was generated for the suspension arm; as for the upper arm, the chosen printing plane is x-z. Then, with the help of Ultimaker^®^ Cura 5.11 software, the slicing operation was carried out, i.e., to define the printing parameters, we obtained the kinematic instructions that the machine (printer) will have to carry out to make the component. The part was created with a sandwich-composite layout that was outlined for this exercise. A material solution was developed based on extensive research into polymer materials used in additive manufacturing (AM) processes. Previous research conducted by the author [41] emphasized the critical importance of understanding the behavior of molten polymer materials. Such understanding is essential for achieving optimal part surface quality and mechanical performance, primarily by reducing material anisotropy (see Figure 12). These improvements were accomplished through a systematic optimization process (see Figure 13).

FDM processes have enabled the development of innovative composite material filaments, such as FormFutura^®^ CarbonFil^®^ (Amsterdam, The Netherlands) This filament consists of a PETG base reinforced with 20% carbon fiber, resulting in an exceptionally stiff material for 3D printing. CarbonFil^®^ offers high-dimensional accuracy and minimal warping during printing. Its unique composition—a blend of the HDglass compound with ultra-light, relatively long carbon fiber strands—yields a filament that is twice as stiff as HDglass and, remarkably, 10% more impact resistant. These features make CarbonFil^®^ stand out among carbon fiber reinforced filaments [42] (see Table 2).

The results of the 3-point bending test provided valuable insights for determining the optimal structural configuration for this application. Three main structural solutions were compared: (1) Transversal Honeycomb, (2) Regular Square Honeycomb with wall reinforcement, and (3) Regular Square Honeycomb without reinforcement. The top-performing configuration was the Transversal Honeycomb with three layers of carbon fiber (CF) reinforcement, Honeycomb—Transversal 191.96 MPa Flexural Stress and Flexural Modulus of 11.11 Gpa. Adding an extra CF layer resulted in a further 50% increase in average flexural stress at yield and a 24% increase in flexural modulus. Table 3 gives a comparison between the achieved mechanical properties of the proposed solution, opposite stock-produced aluminum, steel, and carbon fiber-reinforced PETG with another material selection and AM method found in the literature. Productivity and overall print strength quality can be further improved with the use of new age AM equipment and materials, which ought to increase even microstructures, hence overall mechanical behavior.

**Figure 14:**
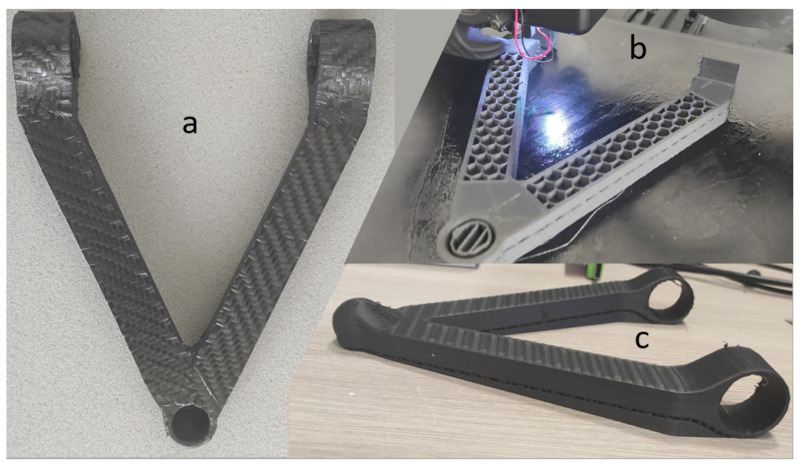
(**a**). Part final laminated version; (**b**). FDM printing of the Upper Arm at 50%; (**c**). AM-produced Arm.

For reasons of cost and time, the prototypes were made to scale: the upper arm was made to scale 1:2. The final, AM version of the arms can be seen in Figure 14b,c, and the laminated upper arm can be seen in Figure 14a.

### Three-Dimensional Scanning: Exploit Data Flow for Quality Control

**Figure 15:**
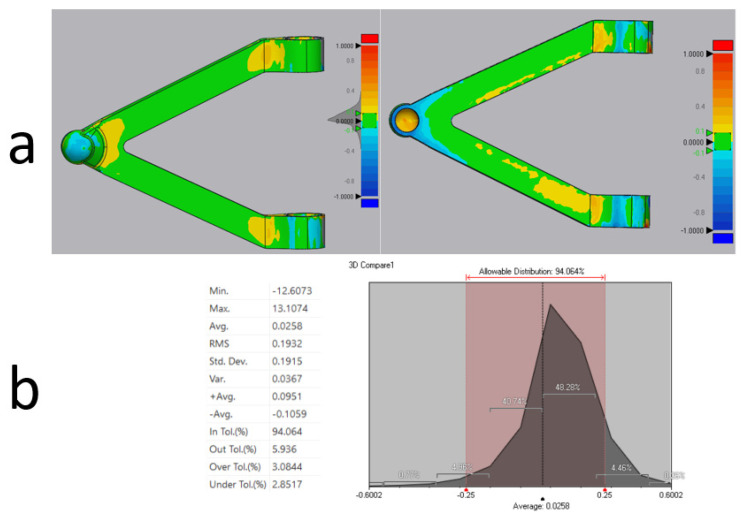
(**a**) Comparison results of scanned part vs. CAD values, (**b**) tolerance variation results by 3D Scanning in Control X 2020.

The last step in prototyping is 3D scanning to verify the surface quality of the print. The scanner builds a point cloud in space, which then through special software, is traced back to the geometry of the printed part. This geometry is then compared with the original CAD geometry from which the part was defined by verifying that the geometric tolerances are within acceptable ranges [Figure 15a]. By scanning the parts, it was found that, for the upper arm, about 94% of the surface area was within a tolerance of ±0.25 mm, while the remaining 6% was beyond this tolerance, specifically 3% above +0.25 mm and the remaining 3% below −0.25 mm [Figure 15b]. This result is considered acceptable because the FDM printing technology used has an extrusion head that produces filaments of 0.4 mm, thus, well below the observed tolerances. In contrast, other AM technologies and materials are capable of reaching narrower tolerance values, like SLM technology within ±0.20 mm tolerance, which is more precise and advanced than FDM. Nevertheless, both prints were found to be acceptable.

## 4. Discussion

The results shown add up to other lightweight AM application exercises [36] in which the goal is to exploit the use of new generation composite materials to respond to other industrial challenges like lowering the overall product lifetime fuel consumption. The use of a structured methodology is valid when overall product variables are taken into account. Designers would follow a method that helps to accurately adapt their product to satisfy different applicability objectives.

### 4.1. Topology Optimization

Previously, a dynamic analysis was conducted in different categories to determine the loads to which the components are subjected. Of this analysis then only the largest loads representative of all situations were considered; these loads represent the input to the topological optimization performed on both arms.

Starting with a simple geometry that considers the footprint of the component within the assembly, this process identified areas where material could be removed without compromising the strength or functionality of the component, resulting in a lighter and more efficient structure geometry for each of the two arms.

### 4.2. Sustainable Prototyping: Slicing and 3D Printing

The upper arm prototype constructed is a 3D-printed, scaled version of the analyzed design seen in Figure 8. This prototype was constructed following the optimized printing process and parameters obtained after following the optimization procedure and nozzle orientation at 0 degrees for best mechanical characteristics [47]; used printing parameters for this part have been optimized by the method proposed in an early study for polymeric FDM [48].

The main objective was to make suspension arms for a vehicle from the original components and optimize them in weight by taking advantage of additive manufacturing. Data on the realization of the optimized arms are shown in Table 4.

From Table 4 it is possible to deduce the advantages and disadvantages related to AM technologies used for prototyping. The values in the table refer to a 1:1 scale prototype. The goal of weight reduction was achieved, especially for the upper arm where a reduction from the original of almost five times was achieved. The trade-off outlined in this study is an extreme lightweighting achievement but at the cost of production time (0.04 pt/h) and with material and process engineering, and it scored an estimated 100,000 cycle lifetime of the proposed design.

The proposed PETG + CF solution is a viable prototype with potentiality for series production. Fatigue performance and productivity have been challenges that continue to improve by offering manufacturability solutions. Material filament proposals are continuing to gain trust for their structural and processability evidence. AM technologies have improved, and now state-of-the-art equipment options can be found on the market with elevated part quality and increased productivity, which can manage to improve overall manufacturing time whilst reducing costs in series production. Quality and repeatability have been the top objective from the main 3D printer companies; the fastest industrial FDM printer currently, the Stratasys F3300 (Minnetonka, MN, USA), stands out for manufacturing by offering double the speed of standard FDM, while FL Sunson T1 Max and high-end Bambu Lab/Creality K1 Series (like K1C) (though more prosumer) push hundreds of mm/s speeds, allowing FDM technology to achieve time and cost effectiveness.

However, more information is needed to completely validate such industrial transformation thoroughly, with the actual need to develop manufacturability and design processes that guarantee the repeatability of using composite structures over traditional material ones. Processability can be tuned up to reduce material anisotropy and optimize surface quality in FDM (see Figure 11), but the trade-off is higher production time. Systematic improvement in such fields will promote the use of these technologies in the industry. Lastly, it is estimated to lower the CO_2_ emissions by 40% per vehicle when overall weight is reduced by 25% [46], contributing to achieving Europe 2035 pollution targets. Lightweight offers manufacturers a flexible method to achieve emissions reduction, without ruling out a path to these emissions savings.

## 5. Conclusions

This study successfully demonstrated that the IDeS-TRIZ framework can guide the design of a suspension arm, optimized for AM. The proposed part resulted in near 85% weight reduction via a proposal of PETG-CF created via FDM and lamination processes whilst maintaining its structural integrity under simulated loads.

The main advantage of using AM was being able to accurately adopt the suggestions given by the topological optimization: this operation often generates very particular geometries because, starting from a starting geometry, it analyzes the stresses in the material and removes parts of material characterized by low stresses. This operation is carried out iteratively according to an algorithm and stops when goals are reached.

A major limitation of these technologies as highlighted by [Table 3] is the production time and sometimes the cost when adopting advanced technologies such as SLM printing.

Slow productivity is inherent in the process that makes the component by adding layer by layer: the fewer layers that are added to the previous one, the more time it takes to make it resulting in increased accuracy of the process.

Companies that want to take advantage of such technologies need new operators who have the skills needed to work with Digital Engineering and AM. AM technology has been the subject of research and development for the past 40 years and has shown that it can create parts of high-quality standards, but despite the wide range of equipment available companies face its high costs. There are still challenges to be faced in terms of quality, productivity, repeatability, and material availability.

Future work will involve the development of a functional full-scale prototype with integrated coupling of metal inserts, experimental dynamic and fatigue testing. Productivity challenges for quality and speed like high-speed sintering or continuous fiber 3D printing could be tested for their applicability.

## Figures and Tables

**Table 1:** Six questions and overall requirements.

SIX QUESTIONS		
**WHO**	What specific sections of the upper and lower wishbones are most subject to stress or wear during normal operation and how do these parts interact and affect the other components of the double wishbone suspension under various loads?	*Joints and small sections (NETWORK STRUCTURES)* *INNOVATION, CUSTOMIZATION*
**WHAT**	What exactly do the upper and lower wishbones represent in a double wishbone suspension and what are the technical specifications, performance and strength expectations needed for these components in the context of the overall functioning of the vehicle?	*Upper arm—COMPRESSION (buckling -> flexion); lower arm—FLEXION* *MECHANICAL STRENGTH, FATIGUE LIFE*
**WHERE**	Where are the maximum loads concentrated on the upper and lower suspension arms during normal driving situations such as braking, cornering, acceleration?	*The applied loads as remote points in the joint centroids > Considered for modeling*
**WHEN**	When during the various phases of vehicle operation (acceleration, braking, braking downhill, cornering) do the upper and lower arms of the double wishbone suspension undergo the maximum stress and the heaviest loads?	*When cornering (conditions explained in 8.2)* *MECHANICAL STRENGTH, DESIGN*
**WHY**	Why is it important to optimize the upper and lower wishbones of the suspension considering aspects such as ride comfort and durability?	*Component Weight Reduction (consequent reduction in inertia)* *MECHANICAL STRENGTH, LIGHTWEIGHT, VERSATILITY*
**HOW**	How are we going to optimize the design of the upper and lower wishbones, in terms of materials, construction techniques or design, to improve their performance, strength and durability in the context of overall vehicle operation?	*Implementation of AM-Optimized design to reduce part density—lattice structures inside the components* *LIGHTWEIGHT, COST, SPEED, EASE, GREEN, AESTHETICS*

**Table 2:** Mechanical properties of CarbonFil^®^.

Properties	Typical Value	Test Method	Test Condition
Mechanical			
Impact strength Tensile strength Tensile modulus Elongation at break Flexural strength Flexural modulus Hardness	5 KJ/m^2^ 92 Mpa 9495 Mpa 3.4% - - -	ISO 179 [43] ISO 527 [44] ISO 527 ISO 527 - - -	Charpy Notched @23 °C @Break - Strain at Break - - -
Main polymer composition	PETG blend		
Reinforcement fiber	Carbon fiber 20%		

**Table 3:** Mechanical properties of different materials.

	PETG + CF	Al	Steel	AM PETG + CF [45,46]
Mechanical				
Impact strength	5 KJ/m^2^	48 KJ/m^2^	30 KJ/m^2^	7 KJ/m^2^
Tensile strength	92 Mpa	140 Mpa	440 Mpa	150 MPa
Tensile modulus	9495 Mpa	69 Gpa	200 Gpa	10.2 Gpa
Flexural stress	191.96 Mpa	240–300 Mpa	370 Mpa	335 Mpa
Flexural modulus	11.11 Gpa	69 GPa	200 GPa	Up to 38 GPa

**Table 4:** What–how matrix.

UPPER ARM					
	Material	Mass [kg]	Cost [EUR]	Technology	Productivity
Original	Steel	2.5	120	Sheet metal working	* 30 pt/h
Optimized	PETG + CF	0.560	380 *	AM FDM	0.04 pt/h

*: Cost includes forming tooling; estimated raw productivity values shown.

## Data Availability

Data associated with this document can be requested from the authors.
